# Emodin induced hepatic steatosis in BALb/c mice by modulating the gut microbiota composition and fatty acid metabolism

**DOI:** 10.3389/fphar.2024.1516272

**Published:** 2024-12-24

**Authors:** Xinhua Xia, Xueling He, Jinzhou Huang, Xuyang Hou, Chen Lin, Yaxiong Liu, Mei Liu

**Affiliations:** ^1^ TCM Department, The First Affiliated Hospital of Guangzhou Medical University, Guangzhou, Guangdong, China; ^2^ Institute of Integrated Chinese and Western Medicine, Guangzhou Medical University, Guangzhou, Guangdong, China; ^3^ Guangdong Provincial Key Laboratory of Research and Development in TCM, Guangdong Second Hospital of Traditional Chinese Medicine, Guangzhou, Guangdong, China; ^4^ The Key Laboratory of Rapid Testing, State Food and Drug Administration, Guangdong Institute for Drug Control, Guangzhou, Guangdong, China; ^5^ School of Agriculture and Biology, Zhongkai University of Agriculture and Engineering, Guangzhou, Guangdong, China

**Keywords:** emodin, polygoni multiflori radix, hepatic steatosis, gut microbiota, FFAs, non-targeted metabolomics

## Abstract

**Introduction:**

The aim of this study is to examine the physiological effects of emodin on intestinal microorganisms and the liver in the BALb/c mice.

**Method and Results:**

Following an 8-week administration of emodin at doses of 25, 50, and 100 mg/kg/day,pathological analyses revealed that emodin significantly reduced the colon length, induced colonic crypt inflammation,diminished the colonic mucus layer,and decreased the fluorescence intensity of colonic tight junction proteins ZO-1 and Occludin. Concurrently, 16S rDNA gene sequencing corroborated that emodin altered the diversity and composition of the intestinal microbiota by increasing the *Firmicutes* to *Bacteroides* ratio. Simultaneously, the non-targeted metabolomics analyses exhibited significant alternations in both short chain fatty acids and free fatty acids between the emodin-treated and the normal groups, indicating emodin-induced disturbance in intestinal metabolic disorder. Furthermore, emodin exhibited a significant elevation in LPS levels in colon, serum and liver as well an marked increase in the levels of TC, TG, AST, and ALT in serum. Additionally, histological examination employing by HE and oil-red O staining furtherly verified that the administration of varying doses emodin induced hepatic inflammation and lipid accumulation. Whereas qRT-PCR and Western blot analyses demonstrated that the administering of varying doses of emodin upregulated the mRNA levels of TNF-α, IL-1β, IL-6, and IL-18 as well as the expression of TLR4, Myd88, and P-65. Following the combined administration of probiotics, the high-dose emodin did not significantly influence ALT and AST levels in mice. However, the faeces of the high-dose emodin transplanted in mice and induced a significant increase in AST levels and in the relative abundance of *Firmicutes* and *Proteobacteria*.

**Discussion:**

These findings further corroborate that emodin induces liver injury via the intestinal dysfunction. These findings suggested that emodin may disrupt intestinal microbiota and resulted in significant alternations in endogenous metabolites in mice, thereby facilitating the entry of LPS and FFAs into the liver, potentially leading to hepatic injury.

## 1 Introduction

Emodin, chemically known as 1,3,8-trihydroxy-6-methylanthraquinone, is a naturally occurring anthraquinone compound extensively identified in various herbal medicines, including Rhei Radix et Rhizoma (Wang, 1993), Aloe (Elsohly et al., 2007), Polygoni Cuspidati Rhizoma et Radix (Qian et al., 2008), Polygoni Multiflori Radix (Liang et al., 2010), and Cassiae Semen (Yang et al., 2014). This compound exhibits several pharmacological properties such as purgative effects, hepatoprotective and choleretic activities, lipid-lowering, antihypertensive, and antioxidant properties (Semwal et al., 2021).

According to the 2020 edition of China Pharmacopoeia, emodin is recorded as the one of critical index components for assessing the quality of Polygoni Multiflori Radix (PMR). It is stipulated that the combined content of emodin and emodin methyl ether should not be less than 0.1%, underscoring emodin as a crucial bioactive constituent responsible for the pharmacological effects of PMR. However, it is particularly noteworthy that reports of adverse reactions have been increasing with the widespread use of PMR in recent years. Most of these adverse reactions are associated with liver injury of varying severity, with mild cases resolving spontaneously upon drug withdrawal, while severe cases may progress to significant liver injury, liver failure, or even death (Xia et al., 2017; Dai et al., 2023). Emodin, as one of the principal active constituents of PMR raise a question of its potential responsibility for liver toxicity. Several studies have identified anthraquinones as the primary hepatotoxic components in PMR (Wang et al., 2022; Xing et al., 2019; Hu et al., 2021).

However, the deleterious impact of emodin on liver, as well as on organs closely associated with the liver such as the intestine, remains uncertain. Given that anthraquinones are primarily absorbed in the intestine, prolonged consumption of PMR as a tonic agent over a period of three to 6 months may result in sustained stimulation of the intestinal mucosa by anthraquinones like emodin. This prolonged exposure may potentially compromise the integrity of the intestinal mucosal barrier. In the event of the intestinal mucosal barrier dysfunction, the increased intestinal permeability permits substantial influx of pathogen-associated factors, such as LPS and other microbial metabolites, into the systemic circulation via the portal vein. This process may exacerbate hepatic inflammation and contributes to liver injury. Consequently, the current study aims to investigate the effects of an 8-week administration of emodin on the intestinal mucosal barrier in mice. Specifically, it examines the impact of varying doses of emodin on the chemical barrier produced by goblet cells, the physical barrier maintained by intestinal endothelial cells, and the biological barrier constituted by the intestinal microbiota. Furthermore, this study seeks to determine the subsequent impact on the liver if emodin induces damage to the intestinal mucosal barrier.

## 2 Materials and methods

### 2.1 Drug and reagent

The emodin compound (Batch No. 201904-2002), with a purity exceeding 98%, was procured from the Shanghai Research Center for Standardization of Traditional Chinese Medicine (Shanghai, China). *Lactobacillus* and *Enterococcus* powder were obtained form Shanghai SINE pharmaceutical Co., LT (Shanghai, China). Primary antibodies targeting ZO-1 and Occludin, along with the secondary antibody of Anti-rabbit Alexa Fluor 488, were sourced from Affinity (Nanjing, Jiangshu, China). Elisa kits for AST, ALT, TC, and TG were obtained from the Nanjing Jiancheng Bioengineering Institute (Nanjing, Jiangshu, China). Polymerase chain reaction (PCR) primers were biosynthesized by Sangon Biotech (Shanghai, China). Hematoxylin and eosin (HE) staining and Oil Red O staining reagents were acquired from Wuhan Seville Biology Co., LT (Wuhan, Hubei, China).

### 2.2 Animal experiment

Thirty-two male BALb/c mice (weighing 18–22 g and aged 6–8 weeks) were purchased from the Experimental Animal Resource Center at Guangzhou Medical University (SCXK 2018-0034). The mice were housed under SPF conditions, maintained at a temperature of 20°C–25°C and a humidity level of 55%–65%, with a 12-h light/dark cycle. They were provided with sterilized water and a standard diet. All experimental procedures were conducted in accordance with the National Institutes of Health Guide for the Care and Use of Laboratory Animals. The present animal experimental protocol received prior approval from the Animal Care Committee of Guangzhou Medical University.

Following a 1-week acclimatization period, the mice were randomly allocated into four distinct groups: the normal group (NC, n = 8), the low-dose emodin group (Emodin-L, n = 8), the medium-dose emodin group (Emodin-M, n = 8), and the high-dose emodin group (Emodin-H, n = 8). The mice in the normal group received an intragastric administration of a CMC-Na solution once daily. The emodin dosage was determined based on our previous study (Xia et al., 2017) as follows: the dosage for mice was established to be 9.1 folds the equivalent of a daily dose of 30 g of Polygoni Multiflori Radix for a 70-kg adult. The extract yield from Polygoni Multiflori Radix was 6.4%. and the concentration of emodin within the extract was 4.2%. Consequently, the emodin dosage for mice was calculated to be 30 g/70 kg × 9.1 % × 1,000% × 6.4 % × 4.2 % = 10 mg/kg/day. Considering that emodin methyl ether present in the extract can be hydrolyzed into emodin upon entering the body. The low, medium, and high doses of emodin in the current study were set as 2.5-fold, 5-fold, and 10-fold, respectively, relative to the equivalent dose of emodin in the extract. In contrast, the mice in the three treatment groups were administered emodin intragastrically at respective doses of 25 mg/kg/day for the low-dose group, 50 mg/kg/day for the medium-dose group, and 100 mg/kg/day for the high-dose group. The treatment lasted over 8 weeks, during which daily monitoring of body weight and general appearance was conducted. Following the final oral administration of varying emodin, blood samples were collected from the retroorbital venous plexus of the mice under anesthesia. Subsequently, all mice were euthanized via cervical dislocation, and a rapid dissection was performed to extract the ileocecal contents, colon, thymus, spleen, and liver. The organs were immediately weighed and then rapidly frozen using liquid nitrogen. Post-freezing, these tissues were stored at −80°C for subsequent analysis.

### 2.3 The quantitative analyses of serum lipids and LPS

The concentrations of AST, ALT, TC, and TG in serum were quantitatively assessed using specific ELISA kits designed for each analyte. All experimental protocols adhered strictly to the manufacturer’s instructions. The absorbance measurements for AST, ALT, TC, and TG were obtained at a wavelength of 510 nm. The sample concentrations, expressed in mmol/L, were calculated using the formula: Sample concentrations (mmol/L) = (Sample OD value - blank OD value)/(Standard OD value - blank OD value) × Standard concentration (mmol/L).

The concentrations of LPS was qualified using the double antibody sandwich method. In this assay, a 96-well plate was initially coated with a mouse-derived LPS antibody as the solid phase antibody. Subsequently, samples or standard LPS along with an HRP-conjugated LPS antibody were sequentially introduced to form an antibody-antigen-enzyme-labeled antibody complex. Following this, TMB was added for color development to facilitate color development, resulting in a color change from blue to yellow. The LPS concentration in the sample was determined by measuring the color intensity of the solution, which is directly proportional to the LPS concentration.

### 2.4 Histopathological examination

Initially, sections of colon and liver tissues underwent HE staining to examine the histopathological alterations induced by varying doses of emodin. The specific procedure for HE staining is as follows: the colon and liver tissues were fixed in 4% paraformaldehyde for a minimum of 48 h, followed by dehydration through a graded ethanol series, and subsequently embedded in paraffin. The paraffin-embedded tissues were then sectioned into 4 μm slices. These sections were stained with hematoxylin and eosin according to the instructions provided with the HE staining kit. Finally, the stained sections were examined under a light microscope (Nikon, Tokyo, Japan). The histological score was determined based on our previous study (He et al., 2021) as follows: the assessment of injury involved summing the scores assigned for the extent of inflammatory cell infiltration, the severity of mucosal damage, and the degree of crypt injury, as outlined below: a score of 0 indicated no evident inflammation, no mucosal damage, and no apparent crypt injury; a score of 1 denoted mild inflammation, mucosal damage, and slight crypt injury; and a score of 2 represented moderate inflammation, damage to both mucosal and submucosal layers, and moderate crypt injury, with only the surface epithelium remaining intact; and a score of 3 exhibited severe inflammation with transmural damage and complete loss of crypts and epithelium.

Subsequently, another portion of liver tissue underwent Oil Red O staining to assess changes in lipid metabolism. Initially, frozen sections of fresh tissue were fixed with formaldehyde calcium for 10 min, followed by rinsing with running water for 1–2 min. The sections were then immersed in 60% isopropyl alcohol before being stained with Oil Red O solution. The staining process continued with differentiation using 60% isopropyl alcohol until the background appeared colorless. Subsequently, the liver nuclei were lightly stained with Mayer’s hematoxylin for 1 min and rinsed with running water for 2 min. The stained liver sections were then observed under the light microscope (Nikon, Tokyo, Japan).

Additionally, Alcian blue (AB) staining was employed to examine alterations in the mucous layer secreted by goblet cells within the colon. The colonic mucous layer serves as an effective chemical barrier by separating intestinal bacteria from the intestinal mucosal epithelial cells. The AB staining procedure was carried out in accordance with the instructions provided in the AB staining kit. In summary, the colon sections were deparaffinized and rehydrated, followed by the application of the AB staining solution for 10 min. Subsequently, the sections were rinsed with running water for 2 min. The nuclei of the colon cells were then lightly counterstained with Mayer’s hematoxylin for 1 min. Finally, the sections were rinsed with running water for 2 min, and all cleaned sections were dehydrated using a recommended gradient alcohol to render them transparent with xylene. Following this process, the sections were sealed with neutral gum and examined under a light microscope (Nikon, Tokyo, Japan).

Furthermore, immunofluorescent staining was conducted to examine the alterations in two critical tight junction proteins, ZO-1 and Occludin. The paraffin-embedded colon sections underwent deparaffinization, followed by dewaxing with xylene and rehydration through a graded alcohol series. Subsequently, antigen retrieval was performed on all sections three times using a microwave and citrate buffer. The sections were then permeabilized with 0.3% Triton™ X-100 and blocked with 5% goat serum for a brief period. The tissue sections were incubated overnight at 4°C with either anti-ZO-1 or anti-Occludin antibodies at a dilution of 1:100. Subsequently, the sections were incubated with Alexa Fluor^®^ 488-conjugated anti-rabbit IgG antibodies at room temperature for 2 h, followed by staining with DAPI for 5 min at room temperature. The sections were then examined using a laser scanning confocal microscope (Carl Zeiss, Oberkochen, Germany).

### 2.5 Fecal bacterial DNA extraction and 16S rDNA gene sequencing

Genomic DNA was extracted from the cecal contents of each sample. The V3 and V4 regions of the 16S rDNA were amplified using specific primers with barcodes. The primer sequences 341F/806R (341F: 5′-CCTACGGGNGGCWGCAG-3′; 806R: 5′-GGACTACHVGGGTATCTAAT-3′) were employed for the PCR amplifications. The PCR protocol included an initial denaturation step at 98°C for 30 s, followed by 35 cycles consisting of denaturation at 98°C for 10 s, annealing at 54°C for 30 s, and extension at 72°C for 45 s. Following amplification, the PCR products were excised and quantified using a fluorometric quantification method. The purified amplified products were subsequently combined in equal proportions. Following this, the sequencing joint was attached, and the sequencing library was constructed. The 16S rDNA gene sequencing of all samples was conducted using the Illumina PE250 platform (Illumina Inc., San Diego, CA, United States) to generate paired-end reads.

Upon obtaining the raw reads from the 16S rDNA gene sequencing, low-quality reads were filtered out, and high-quality reads were assembled and re-filtered to acquire valid data for operational taxonomic unit (OTU) clustering. This process utilized the Usearch tool within the QIIME software (version 7.1), employing a 97% sequence similarity threshold to determine the abundance of OTUs. Following the acquisition of OTUs, species annotation, alpha diversity analysis, beta diversity analysis, and community function prediction were conducted using Maffi software (version 7.310). The compositional structure of the intestinal microbiota was characterized using multivariate statistical methods of principal component analysis (PCA), principal coordinate analysis (PCoA), and non-metric multidimensional scaling (NMDS). Additionally, the LDA Effect Size (LefSe) analysis was employed, as referenced from http://huttenhower.sph.harvard.edu/galaxy/.

### 2.6 Non-targeted metabolomics analyses of the serum

The endogenous metabolites in serum were quantitatively analyzed by an ultra-performance liquid chromatography coupled to tandem mass spectrometry (UPLC-MS/MS) system using the Q300 kit (Metabo-Profile Biotechnology, China, shanghai) at both ion mode of positive and negative. The original data matrix with the information of peak retention time (RT), mass-to-charge ratio (M/Z), peak identity, peak areas was generated by MassLynx software in UPLC-MS/MS to integrate peak, calibrate and quantify each metabolite. After peak alignment and peak filtering, all data were was set as the raw data matrix in order to enable the comparison of data by multivariate statistical analysis like PCA, PLS-DA, OPLS-DA, random forest, support vector machine learning and univariate statistical analysis, Student-t test, Mann- Whitney-Wilcoxon (U-test), variance analysis and correlation analysis at the differing magnitudes.

### 2.7 Real-time quantitative PCR and Western blot analyses

Real-time quantitative PCR analysis was performed to determinate the mRNA levels of inflammatory factors like TNF-α, IL1β, IL6, IL18, TLR4, Myd88, and P-65. The target gene sequence was derived from the mRNA coding region on PubMed and synthesized by Shanghai Sangon Bioengineering Co., LTD. The specific primers utilized in this study are as follows: TNF-α-Forward (5′-CGC​TGA​GGT​CAA​TCT​GC-3′), TNF-α-Reverse (5′-GGC​TGG​GTA​GAG​AAT​GGA-3′), IL-1β-Forward (5′-TTG​AGT​CTG​CCC​AGT​TCC-3′), IL-1β-Reverse (5′-TTT​CTG​CTT​GAG​AGG​TGC​T-3′), IL-6-Forward (5′-CAA​TAA​CCA​CCC​CTG​ACC-3′), IL-6-Reverse (5′-GCG​CAG​AAT​GAG​ATG​AGT​T-3′), IL-18-Forward (5′-TTG​TCT​CCC​AGT​GCA​TTT​T-3′), IL-18-Reverse (5′-GGT​TCC​TTT​CCT​CTT​CCC-3′), TLR4-Forward (5′-ATT​TCC​GCT​TCC​TGG​TCT-3′), TLR4-Reverse (5′-GTC​ATC​CCA​CTT​CCT​TCC​T-3′), Myd88-Forward (5′-CCG​CCT​GTC​TCT​GTT​CTT-3′), Myd88-Reverse (5′-GTC​CGC​TTG​TGT​CTC​CA-3′), p65-Forward (5′-ATG​CGC​TTC​CGC​TAC​AA-3′), and p65-Reverse (5′-GTG​ACC​AGG​GAG​ATG​CG-3′). The standard curve was generated through real-time quantitative PCR amplification. The amplification efficiency was determined base on the slope k of the standard curve, using the formula: amplification efficiency = 10^1/(−k)^−1, where k ranges from −3 to −3.5, amplification efficiency ranges between 0.9 and 1.1. When the amplification efficiencies of the target gene and the reference gene was equivalent (i.e., the difference in their slopes was less than 0.2), the relative expression of the target gene was calculated using the mean relative content = 2^−△△Ct^.

Western blot analysis was conducted to determine the levels of those key proteins closely associated with the inflammation reaction in liver. The frozen liver tissue blocks were homogenized in ice with 10 times the tissue volume of the extraction reagent (add the protease inhibitor within a few minutes before use). After this homogenization, the homogenate was transferred to a 1.5 mL EP tube and oscillate in ice bath for 30 min, the pipette was used to blow the homogenate repeatedly to ensure complete cell lysis. Finally, the homogenate was centrifugated at 12,000 rpm/min (4°C) for 10 min, the supernatant of the total protein solution was collected to normalize the total protein concentrations with a BCA assay kit. After normalization, all protein samples were separated by sodium dodecyl sulfate polyacrylamide gel electrophoresis and transferred to polyvinylidene fluoride membranes. The membranes were blocked for 2 h at room temperature with 5% bovine serum albumin. The blocked membranes were initially incubated overnight at 4°C with primary antibodies, followed by a 1-h incubation at room temperature with the corresponding secondary antibodies. The primary antibodies sourced from Cell Signaling Technology, included TLR4, Myd88, P65, p-P65, and GAPDH (Beverly, MA, United States). Protein expression was detected in accordance with the protocol provided by the Tanon ECL detection system (Shanghai, China). Image acquisition and analysis were conducted using the Kodak 4000RPRO gel imaging system (Rochester, NY, United States).

### 2.8 Animal studies on the application of probiotics and fecal transplantation

In this study, we subsequently conducted animal trials to validate the hepatotoxic effect induced by emodin via intestinal tract.

Initially, probiotics were employed to mitigate emodin-induced hepatic injury in the intestines. Mice were randomly allocated into four groups, each consisting of eight mice: a normal control group and three experimental groups receiving probiotics in combination with emodin at low, medium, and high doses (Probiotics at 1 × 10^9^ CFU/kg/day combined with emodin at 25, 50, and 100 mg/kg/day, respectively). All treatments were administered via gavage over an 8-week period. Upon conclusion of the experiment, serum samples from the mice were collected to assess liver functional indicators, including AST, ALT, and ALP.

Fresh fecal samples from mice in the low and high-dose emodin groups were collected and weighed following the intragastric administration. The fecal samples from each group were then dissolved in normal saline at room temperature, adhering to a mass to volume ration of 1:5. After thorough mixing, larger insoluble particles were filtered out using three layers of gauze repeatedly. The fecal bacterial suspension was initially collected and measured using a 10-mL centrifuge tube to prepare the preliminary fecal bacterial solution. This suspension was subsequently centrifuged at 1,200 rpm/min for 3 min. Following centrifugation, the supernatant was discard, and the normal saline was added to restore the volume to its original level. The mixture was then thoroughly agitated and subjected to centrifugation again. This process was repeated a total of three times to isolate the sediment. The resultant sediment was then dissolved in normal saline to achieve a concentration of 200 mg/mL and was administered to the low-dose (n = 6-7, Faeces-Low-dose) and high-dose (n = 6, Faeces-high-dose) of fecal bacteria transportation groups via gavage for 8 week. At the end of experiment, all mice were anesthetized to facilitate the collection serum and the ileocecal contents for further analyses.

### 2.9 Statistical analysis

In this study, statistical analyses were conducted using SPSS software version 23.0, while graphical representations were generated with GraphPad Prism version 9.0. Experimental data are presented as mean ± SD, contingent upon their conformity with a normal distribution. For multivariate comparisons, a one-way analysis of variance (ANOVA) was employed, followed Tukey’s *post hoc* test or Student’s t-test, as appropriate. Statistical significance was determined at *p* < 0.05 (*), *p* < 0.01 (**), *p* < 0.001 (***), and *p* < 0.0001 (****).

## 3 Results

### 3.1 The administration of varying doses of emodin over an 8-week period significantly impacted the overall physical condition of the mice

**FIGURE 1:**
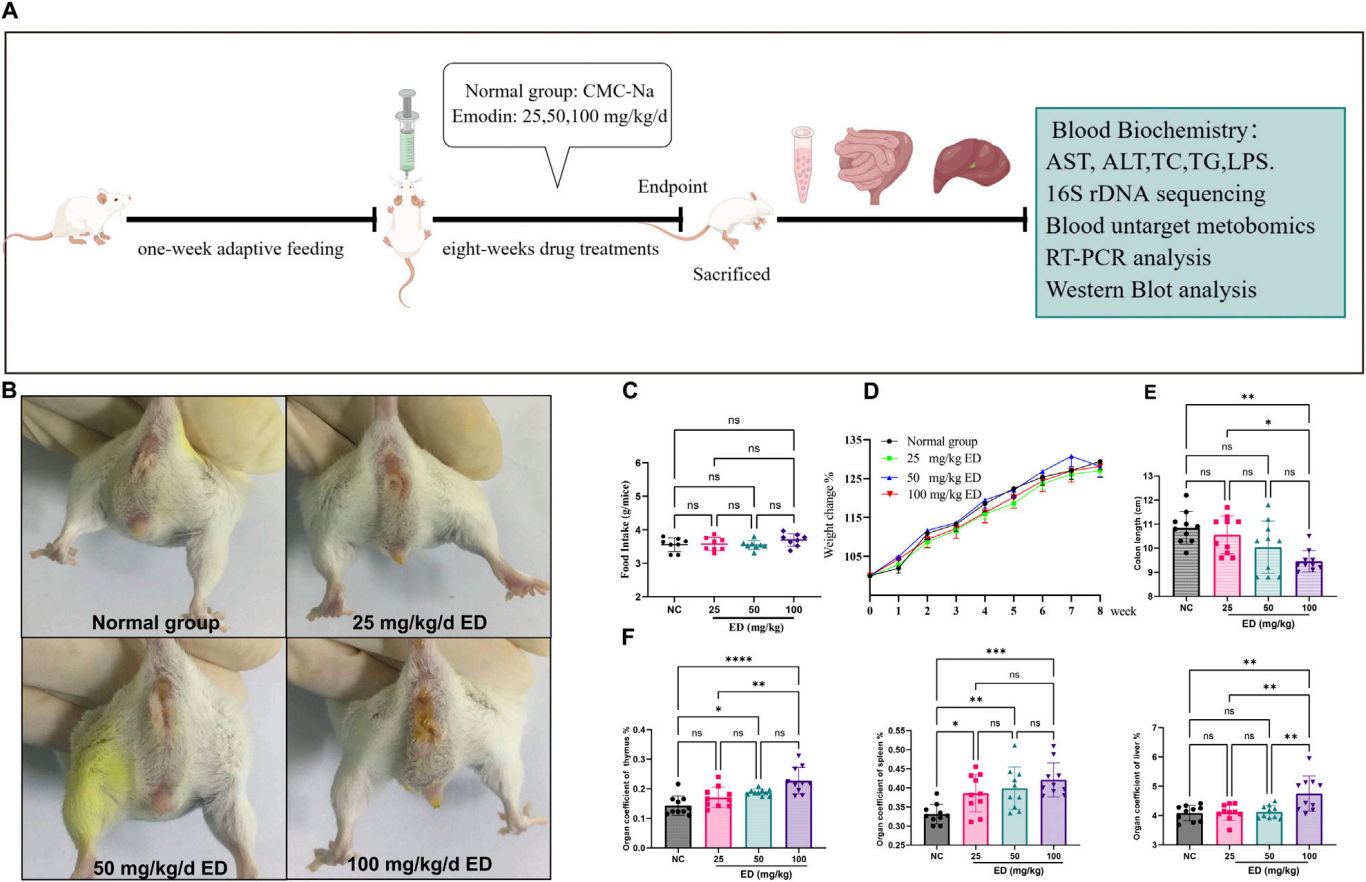
Observational results regarding the overall condition of mice following 8 weeks of administration of varying doses of emodin: **(A)** Experimental design for the animal study; **(B)** Presence of loose or watery stools around the anal region of mice after 8 weeks of emodin treatment; **(C)** Comparative analysis of food intake among the four groups of mice; **(D)** Weight changes observed in the four groups of mice throughout the experimental period; **(E)** Comparison of colon lengths among the four groups; **(F)** Analysis of organ coefficients for the thymus, spleen, and liver across the four groups. Statistical significance is indicated as follows: *p < 0.05, **p < 0.01, ***p < 0.001, and ****p < 0.0001.

The overall design of the animal experiment is illustrated in Figure 1A. At the conclusion of the study, mice in three treatment groups exhibited characteristic abnormal behaviors, including coarse fur, hyperactivity, aggression, and diarrhea. Notably, the incidence and frequency of diarrhea were higher in the groups receiving emodin doses of 50 and 100 mg/kg/day compared to both the normal group and the low-dose group. Furthermore, a small number of mice administered a 100 mg/kg/day of emodin developed bloody stools and mild prolapse (Figure 1B). Although the majority of mice in three treatment groups developed loose stools, this condition did not significantly affect their food intake or weight (Figures 1C, D). Furthermore, upon dissection of various organs from the mice, it was observed that varying doses of emodin reduced the colon length. As illustrated in Figure 1E, the doses of 25 and 50 mg/kg/day of emodin resulted in a reduction in colon length without reaching statistical significance compared to that in the normal group. However, a dose of 100 mg/kg/day of emodin significantly shortened the colon length (*p* < 0.01). Meanwhile, Figure 1F revealed that the coefficients of the spleen, thymus, and liver exhibited a dose-dependent increase. In comparison to the normal group, all three treatment doses of emodin led to a significant increase in the spleen coefficient (*p* < 0.05, *p* < 0.01, *p* < 0.001, respectively). However, only the 50 and 100 mg/kg/day doses of emodin significantly elevated the thymus coefficient (*p* < 0.05, *p* < 0.0001), while the 25 mg/kg dose resulted in a slight, non-significant enhancement. Additionally, the liver coefficient was significantly increased only at the 100 mg/kg/day dose of emodin (*p* < 0.01).

### 3.2 The administration of varying doses of emodin over an 8-week period resulted in remarkable damage to the intestinal mucosal barrier in the mice

**FIGURE 2:**
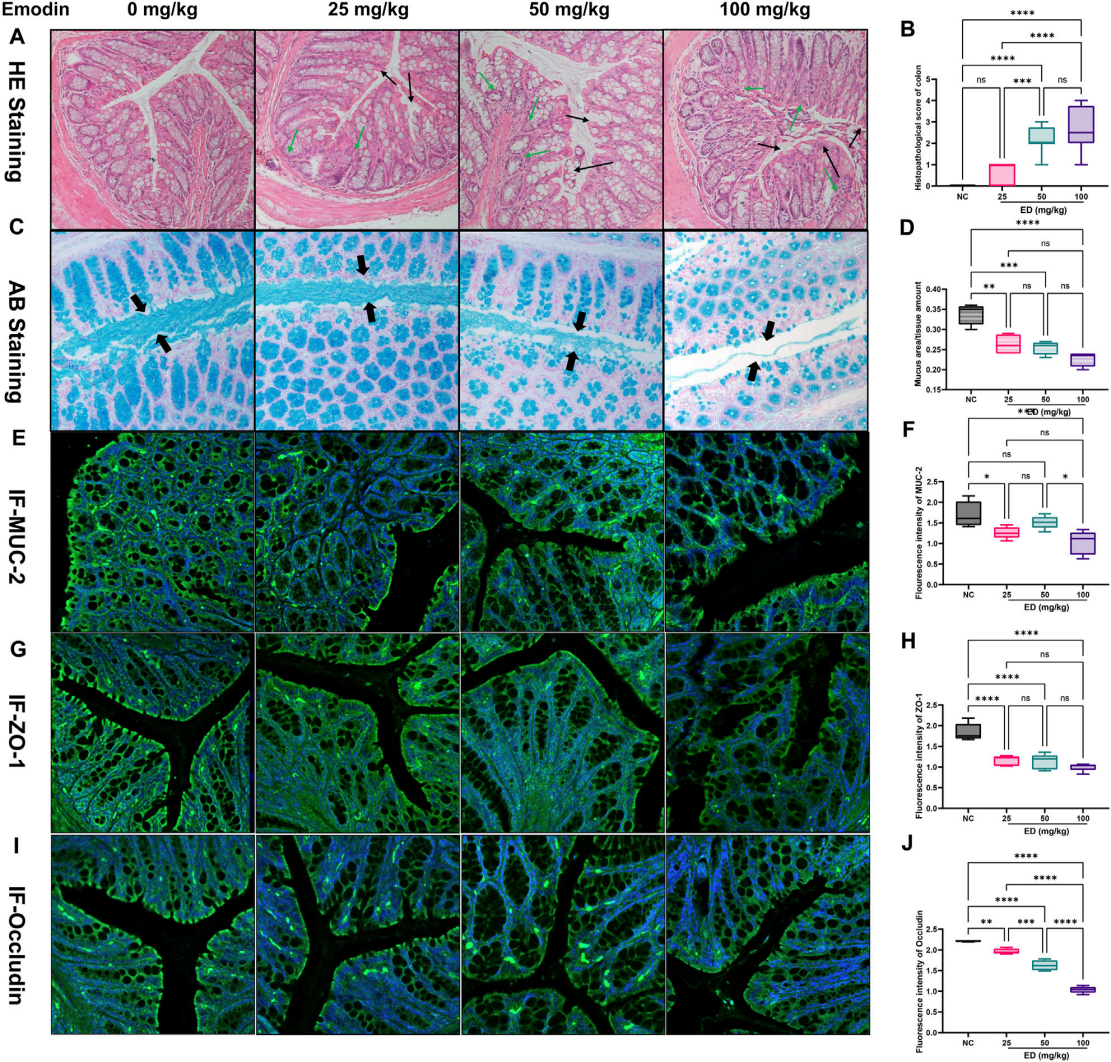
Histopathological examination results of the intestinal tract in mice at the experimental endpoint: **(A)** Representative HE stained images of colon from four experimental groups; **(B)** Comparative analysis of HE scores across the four groups; **(C)** Representative AB stained images illustrating the mucus layer secreted by goblet cells in the four groups; **(D)** Comparative analysis of mucus area-to-issue amount ratio in the four groups; **(E)** Representative immunofluorescent images of MUC-2 expression in the colon across the four groups; **(F)** Comparative analysis of MUC-2 immunofluorescent intensity among the four groups; **(G)** Representative immunofluorescent images of ZO-1 in the colon across the four groups; **(H)** Comparative analysis of ZO-1 immunofluorescent intensity among the four groups; **(I)** Representative immunofluorescent images of Occludin in colon for the four groups; **(H)** Comparative analysis of Occludin immunofluorescent intensity among the four groups. Statistical significance is indicated as follows: *p < 0.05, **p < 0.01, ***p < 0.001, and ****p < 0.0001.

As demonstrated in the HE staining images in Figure 2A, the cellular morphology of colonic epithelial cells exhibited significant alterations with increasing doses of emodin. Specifically, administration of 25 mg/kg/day of emodin exerted minimal impact on the cellular morphology, with cells appearing intact and comparable to those in the normal group. In contrast, the 50 mg/kg/day of emodin displayed obvious evidence of colonic mucosal injury and mild inflammatory infiltration. More pronounced colonic injury, characterized by partial degeneration, necrosis of the colonic mucosa, and marked inflammation, was induced by the 100 mg/kg/day dose of emodin. Furthermore, the HE staining scores for the three emodin groups were 0.625, 2.125, and 2.625, corresponding to increasing doses of emodin (refer to Figure 2B). The scores administering 50 and 100 mg/kg/day of emodin were significantly higher than those of the normal group (*p* < 0.001, *p* < 0.0001).

Subsequently, additional functional indicators associated with the integrity of the intestinal mucosal barrier were examined using alternative staining methods. For example, Goblet cells primarily secrete MUC-2 mucin, which in conjunction with water, inorganic salts, and antimicrobial peptides in the intestinal tract, forms the viscous gel-like reticular mucus layers that serve to protect epithelial cells. As shown in the representative images from Figure 2C, it revealed that the thickness of the blue mucous layer was significantly reduced in the group administered with 25 mg/kg/day dose of emodin compared to the normal group. This reduction was more pronounced in the groups receiving 50 and 100 mg/kg/day dose of emodin. Concurrently, the ratio of mucous area to total tissue volume, as depicted in Figure 2D, corroborated these observations, showing a marked decrease to 76.5% (25 mg/kg/day of emodin, *p* < 0.01), 73.5% (50 mg/kg/day of emodin, *p* < 0.001), and 67.6% (100 mg/kg/day of emodin, *p* < 0.0001) relative to the normal group. Moreover, MUC-2 is a predominant component of mucinous glycoprotein involved in the formation of the mucous layer. The relative expression levels of MUC-2 were assessed through immunofluorescent staining, as depicted in Figure 2E. The analysis revealed that the green fluorescence intensity of MUC-2 in the three treatment groups decreased to 73.9% (25 mg/kg/day of emodin, *p* < 0.05), 88.9% (50 mg/kg/day of emodin, not significant), and 60.8% (100 mg/kg/day of emodin, *p* < 0.01), Figure 2F relative to the normal group.

In addition to the protective role of the outer mucus layer, tight junction proteins such as ZO-1 and Occludin are crucial for maintaining intestinal barrier integrity. Abnormal localization and expression of ZO-1 and Occludin can directly affect the permeability of intestinal endothelial cells. As demonstrated in Figure 2G, the yellow-green fluorescence intensity of the ZO-1 protein progressively diminished with increasing doses of emodin. Specifically, compared to the normal group, the fluorescence intensity of ZO-1 was significantly reduced in the emodin-treated groups, with values decreasing to 62.8% in the low-dose of emodin group (25 mg/kg/day, *p* < 0.0001), 61.2% in the medium-dose of emodin group (50 mg/kg/day, *p* < 0.0001), and 54.4% in the high-dose of emodin group (100 mg/kg/day, *p* < 0.0001) (refer to Figure 2H). Similarly, the yellow-green fluorescence of Occludin exhibited a dose-dependent reduction across the three treatment groups (refer to Figure 2I), with fluorescence intensity decreasing by 11.5% (*p* < 0.01), 26.5% (*p* < 0.0001), and 53.2% (*p* < 0.0001) in the 25, 50, and 100 mg/kg/day doses of emodin groups, respectively, compared to the normal group (Figure 2J).

### 3.3 The administration of varying doses of emodin over an 8-week period led to dysbiosis of the in the mice

A total of 32 ileocecal samples were analyzed using 16S rDNA gene sequencing, focusing on the V3 and V4 regions across four treatment groups. This analysis yielded 3,118,287 pairs of reads and 3,193,620 clean tags for all samples. The clean tags were then clustered by removing chimeric tags to obtain effective tags.

**FIGURE 3:**
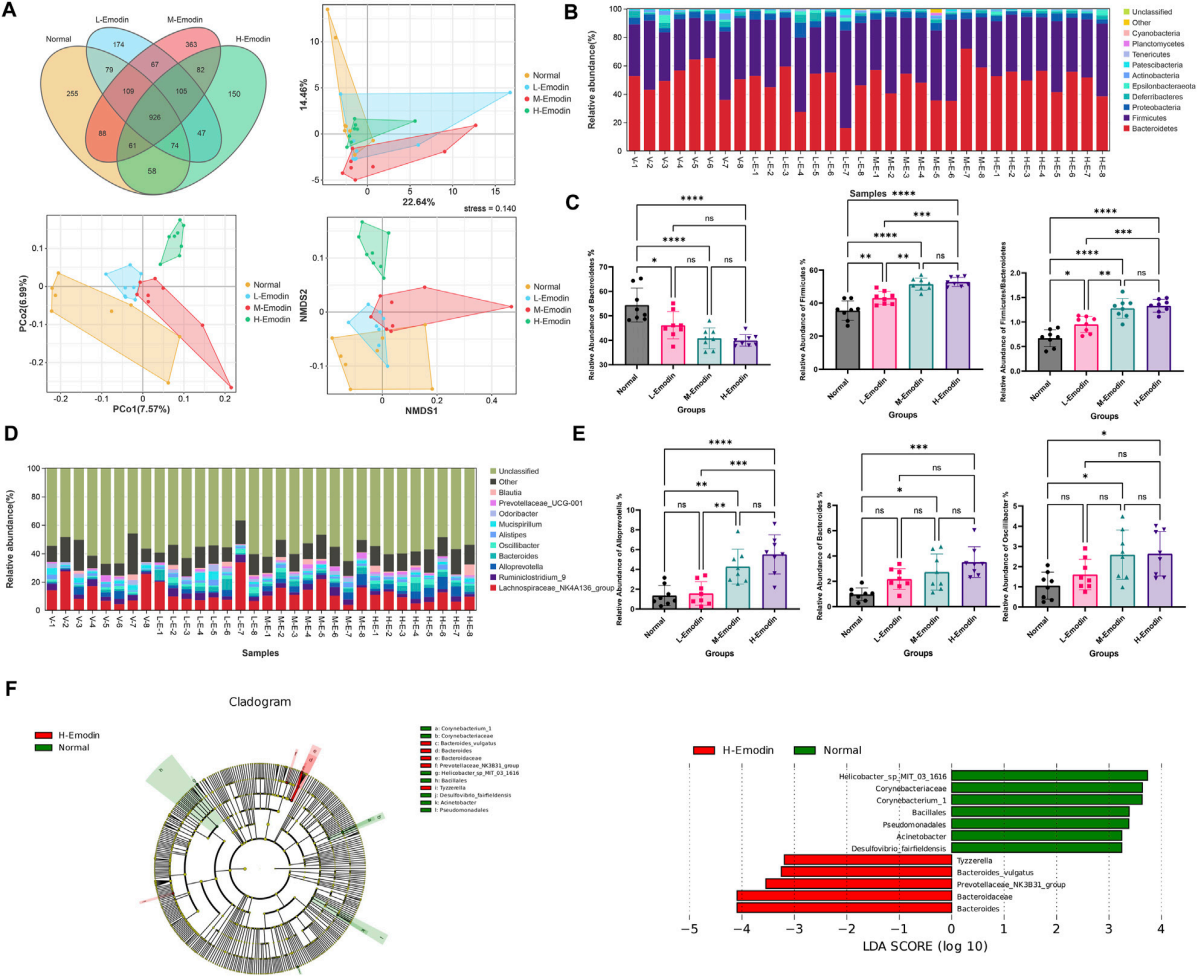
Emodin induced the disruption of gut microbiota: **(A)** the Venn’s analysis and multivariate statistical analysis including PCA, PCo2, and NMDS among four groups based on the OUTs; **(B)** A histogram depicting the identified gut microbiota phyla across samples; **(C)** A comparative analysis of the relative abundance of *Bacteroidetes,* and *Firmicutes* as well as *Firmicutes* to *Bacteroidetes* ratio among the four groups; **(D)** A histogram showing the identified gut microbiota genera across samples; **(E)** A comparative analysis of the relative abundance of *Alloprevotella*, *Bacteroides*, and *Oscillibacter* among the four groups; **(F)** LEfSe analysis of the gut microbiota between the normal group and the high-dose of emodin group (the LEfSe analysis identified taxa with significant exhibited differential abundant, characterized by a *p*-value of less than 0.05 and an LDA score exceeding 2.0.). Significance levels are denoted as follows: *p < 0.05, **p < 0.01, ***p < 0.001, and ****p < 0.0001.

Subsequently, these effective tags underwent OTU cluster analysis to generate OTU sequences. Venn’s figure analysis was conducted based on the OTU abundance to elucidate the differences in OTUs between the normal group and those emodin treatment groups. As illustrated in Figure 3A. Specifically, the low, medium, and high-doses of emodin groups shared 1,188, 1,184, and 1,119 common OTUs with the normal group, respectively. Additionally, these groups exhibited 393, 617, and 384 unique OTUs, respectively. Utilizing these common and unique OTUs, advanced PCA, PoCA, and NMDS were performed to investigate the variations in intestinal microbial community composition induced by the varying doses of emodin. As presented in Figure 3A, three emodin treatment groups demonstrated clear differentiation from one another, with samples within each group predominantly clustering in the same region. Specifically, in the PCA image, the plots representing the three emodin groups were primarily situated in the lower right quadrant relative to the plots from the normal group. Conversely, in the PCoA and NMDS images, the plots from the three emodin groups were entirely located in the upper right quadrant compared to those from the normal group. This shift indicates that the composition of the intestinal microbial community was influenced to some extent by varying doses of emodin.

Moreover, additional analyses were conducted to investigate which microbial species at phylum and genus level were significantly affected by varying doses of emodin. As illustrated in Figure 3B, the intestinal microbiota in samples from each group predominantly comprised *Bacteroidetes*, *Firmicutes* and *Proteobacteria*. The administration of emodin influenced the relative abundance of these three phyla bacteria in distinct ways. Specially, the relative abundance of *Bacteroidetes* decreased while that of *Firmicutes* and *Proteobacteria* increased with higher doses of emodin. In particular, the relative abundance of *Bacteroidetes* in the high-dose of emodin group was reduced to an half of that observed in the normal group (Figure 3C)*.* Firmicutes and Bacteroidetes are two predominant bacterial phyla in the human gut microbiota. Numerous studies have demonstrated that *Firmicutes* to *Bacteroidetes* (F/B) ratio is closely associated with obesity and other diseases. In this study, with a decrease in *Bacteroidetes* and a increase in *Firmicutes,* it was observed that emodin administration at low, medium, and high doses significantly increased the F/B ratio by 1.3-fold (*p <* 0.05), 1.7-fold (*p <* 0.0001), and 1.9-fold (*p <* 0.0001), respectively, compared to the normal group (Figure 3C). Furthermore, three doses of emodin altered the composition of the intestinal microbiota at the genus level, affecting taxa such as Lachnospiraceae_NK4A136_group, Ruminiclostridium_9, Alloprevotella, *Bacteroides*, Oscillibacler, Alistipes, Mucispirillum, Odoribacter, Prevotellaceae _UCG_001, and Blautia. Certain bacterial groups, such as Lachnospiraceae_NK4A13 6_group and Ruminiclostridium_9, exhibited a decrease, while others including Alloprevotella and *Bacteroides*, showed an increase compare to the initial phase (Figure 3D). Notably, the relative abundance of Alloprevotella, Bacteroidesin, and Oscillibacler increased significantly in this study. Specially, in the middle-dose emodin group, these increases were over 2.8-fold (*p* < 0.01), 3.1-fold (*p* < 0.05), and 2.5-fold (*p* < 0.05), respectively. In the high-dose emodin group, the increase were over 3.8-fold (*p* < 0.0001), 3.5-fold (*p* < 0.001), 2.5-fold (*p* < 0.05) compared to the normal group (Figure 3E).

Simultaneously, the LeFSe analysis was employed to identify the principal differences in intestinal flora taxa, aiming to ascertain the indicator genera associated with the effects of emodin. At the genus level, potential biomarkers distinguished the normal group from the high-dose emodin group were identified using Lefse analysis (LDA>2), as depicted in histogram and branching diagram in Figure 3F. The findings revealed an enrichment of *Bacteroides*, Prevotellaceae_NK3B31_group, and Tyzzerella in the high-dose emodin group. Among these three potential indicator genera, *Bacteroides*, and Prevotellaceae_NK3B31_group are classified within Bacteroidetes phylum, while Tyzzerella is categorized to *Firmicutes* phylum.

### 3.4 The administration of varying doses of emodin induced the fatty liver hepatitis

**FIGURE 4:**
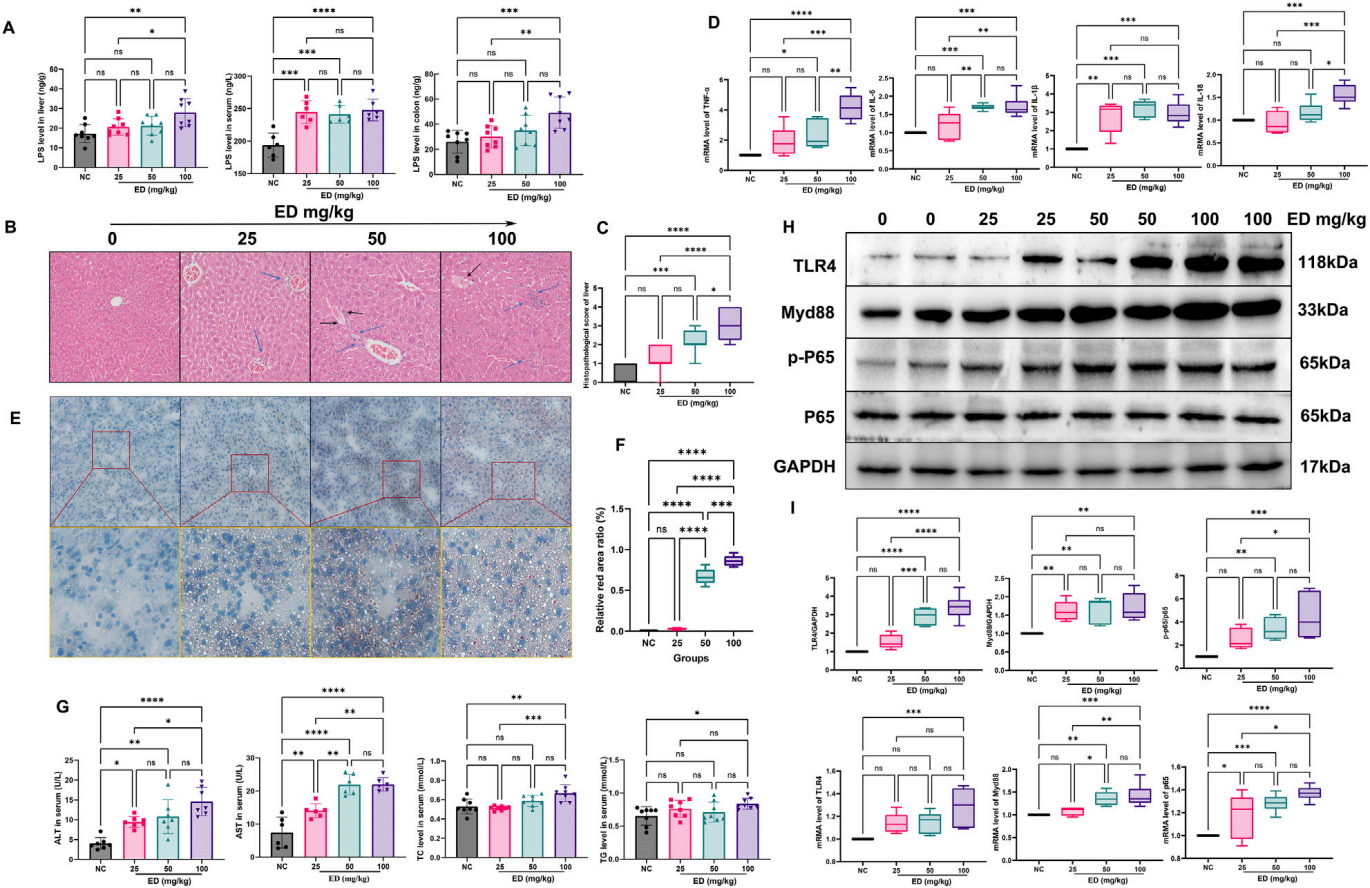
Emodin induced hepatic steatosis in mice: **(A)** Comparison of LPS levels in the liver, blood, and colon across the four groups; **(B)** Representative images of HE staining of liver tissues from the four groups; **(C)** Comparison of HE scores among the four groups; **(D)** Comparison of the mRNA levels of TNF-α, IL-6, IL-1β, and IL-18 in liver tissues among the four groups; **(E)** Representative images of Oil-red staining of liver tissues from the four groups; **(F)** Comparison of the relative red ratio in liver tissues among the four groups; **(G)** Comparison of ALT, AST, TC, and TG levels in mouse serum across the four groups; **(H)** Representative protein bands patterns for TLR4, MyD88, p-P65, and P-65; **(I)** Comparison of the relative proteins expression and mRNA levels of TLR4, MyD88, p-P65, and P-65. Statistical significance is denoted as follows: *p < 0.05, **p < 0.01, ***p < 0.001, and ****p < 0.0001.

LPS, a gut microbial antigen derived from the outer membrane of gram-negative bacteria that triggers secretion of inflammatory cytokines. when the integrity of the intestinal mucosal barrier is compromised, there is an increased permeability to LPS, allowing substantial amounts to traverse the barrier, subsequently reaching the liver and precipitating hepatic inflammation. As illustrated in Figure 4A, the levels of LPS in the colon, serum and liver of three emodin treatment groups were significantly higher than those in the normal group. Notably, the serum LPS levels in the medium and high doses emodin groups exhibited a significant increase (*p* < 0.05 and *p* < 0.01, respectively) compared to the normal group. Furthermore, it is noteworthy that the LPS content in the colon and liver of the high-dose emodin group increased by 2.1-fold (*p* < 0.05) and 1.5-fold (*p* < 0.01), respectively, relative to the normal group. These findings align with the observation that inflammatory infiltration was prominently present in HE stained sections of liver tissue from both the medium and high-dose emodin groups (Figure 4B). Additionally, the medium-dose emodin group displayed a minor hepatic steatosis, while the high-dose emodin group showed an severe inflammatory infiltration and the presence of lipid vacuoles in the liver tissue. Hepatic HE scores demonstrated a dose-dependent increase. Specially, the HE scores for the low-dose emodin group showed an increase without reaching statistical significance. However, they increased by up to 5.6-fold (*p* < 0.05) and 8.3-fold (*p* < 0.001) in the medium-dose and high-dose emodin groups, respectively, compared to the normal group (Figure 4C).

Furthermore, during hepatic inflammation, significant alternations were observed in the levels of inflammatory mediators such as TNF-α, IL-6, IL-1β, and IL-18 both in the liver and bloodstream. Specially, emodin was found to enhance the mRNA expression levels of TNF-α, IL-6, IL-1β, and IL-18 within the liver (refer to Figure 4D). Administration of three different doses of emodin resulted in a significant upregulation of IL-1β mRNA levels (*p* < 0.01, *p* < 0.001, *p* < 0.001, respectively). Additionally, the medium and high doses of emodin markedly increased the mRNA levels of TNF-α (*p* < 0.05, *p* < 0.0001) and IL-6 (*p* < 0.001, *p* < 0.0001). However, only a high dose of emodin significantly increased the mRNA level of IL-18 (*p* < 0.001). Numerous studies have indicated that LPS can induce an inflammatory response by activating the Toll-like receptor 4 (TLR4) signaling pathway, thereby promoting liver inflammation and fibrosis (Engelmann et al., 2020; Schneider et al., 2022). The primary effects of emodin on the upstream proteins such as TLR4, Myd88, P-65 were presented in Figures 4H, I, three different doses of emodin were shown to upregulate the relative expression levels of TLR4, MyD88 and P-65 within the TLR4 pathway in liver tissues. The relative expression of TLR4 increased by 1.5-fold in the lose-dose emodin group, 2.91-fold in the medium-dose emodin group (*p* < 0.0001), and 3.41-fold in the high-dose emodin group (*p* < 0.0001) compared to the normal group. Concurrently, the administration of emodin at three doses elevated the relative expression of Myd88 by 1.62-fold (*p* < 0.01), 1.67-fold (*p* < 0.01), and 1.72-fold (*p* < 0.01) in a dose-independent manner. Furthermore, the medium and high doses of emodin significantly enhanced the phosphorylation level of protein P-65. Specifically, the ratio of p-P-65/P-65 increased to 2.49-fold in the lose-dose emodin group, 3.39-fold (*p* < 0.01) in the medium-dose emodin group, and 4.47-fold (*p* < 0.001) in the high-dose emodin group, respectively, relative to the normal group. Administration of emodin at three doses of emodin resulted in significant upregulation of P-65 mRNA levels, with an increase of 1.18-fold in the lose-dose emodin group (*p* < 0.05), 1.28-fold in the medium-dose emodin group (*p* < 0.001), and 1.37-fold in the high-dose emodin group (*p* < 0.0001). Additionally, two treatments with emodin significantly elevate MyD88 mRNA levels by 36.4% in the medium-dose group (*p* < 0.01) and 42.2% in the high-dose emodin group (*p* < 0.001) compared to the normal group. Notably, only the high dose of emodin was found to significantly upregulate TLR4 mRNA levels, with a 1.28-fold increase (*p* < 0.001).

Despite the observation of emodin-induced liver inflammation, it is important to highlight the concurrent accumulation of fatty acids during the period of emodin administration. As illustrated in Figure 4E, the area represented by the red color (indicating lipid) became increasing prominent. For instance, liver tissue from the low-dose emodin group exhibited only a small number of vacuoles. In contrast, the relative fat area increased by 7.3-fold in the medium-dose emodin group and 9.3-fold in the high-dose emodin group compared to the normal group (Figure 4F). With the elevation of hepatic lipid deposition, there was a significant increase in the levels of TG and TC in the blood. Specifically, administration of a high-dose emodin resulted in a 26.2% increase in TC (*p* < 0.01) and a 28.4% increase in TG (*p* < 0.05) compared to the normal group (Figure 4G). Additionally, emodin also facilitated a dose-dependent increase in serum AST and ALT in mice. The AST levels were 1.9-fold (*p* < 0.01) higher with the low-dose emodin, 2.5-fold (*p* < 0.0001) with the medium-dose emodin, and 2.9-fold (*p* < 0.0001) with the high-dose emodin, relative to the normal group. During the study, the ALT levels increased 2.4-fold (*p* < 0.01, the low-dose emodin), 2.7-fold (*p* < 0.01, the medium-dose emodin), and 3.6-fold (*p* < 0.0001, the high-dose emodin) compared to the normal group.

### 3.5 An 8-week administration of varying doses of emodin significantly disrupted fatty acid metabolism in the blood of mice

Liver, being the primary organ responsible for lipid metabolism, was notably affected by varying dose of emodin. This study further employed non-targeted metabolomics to metabolically characterize the lipid metabolism disorder induced by emodin in the blood.

**FIGURE 5:**
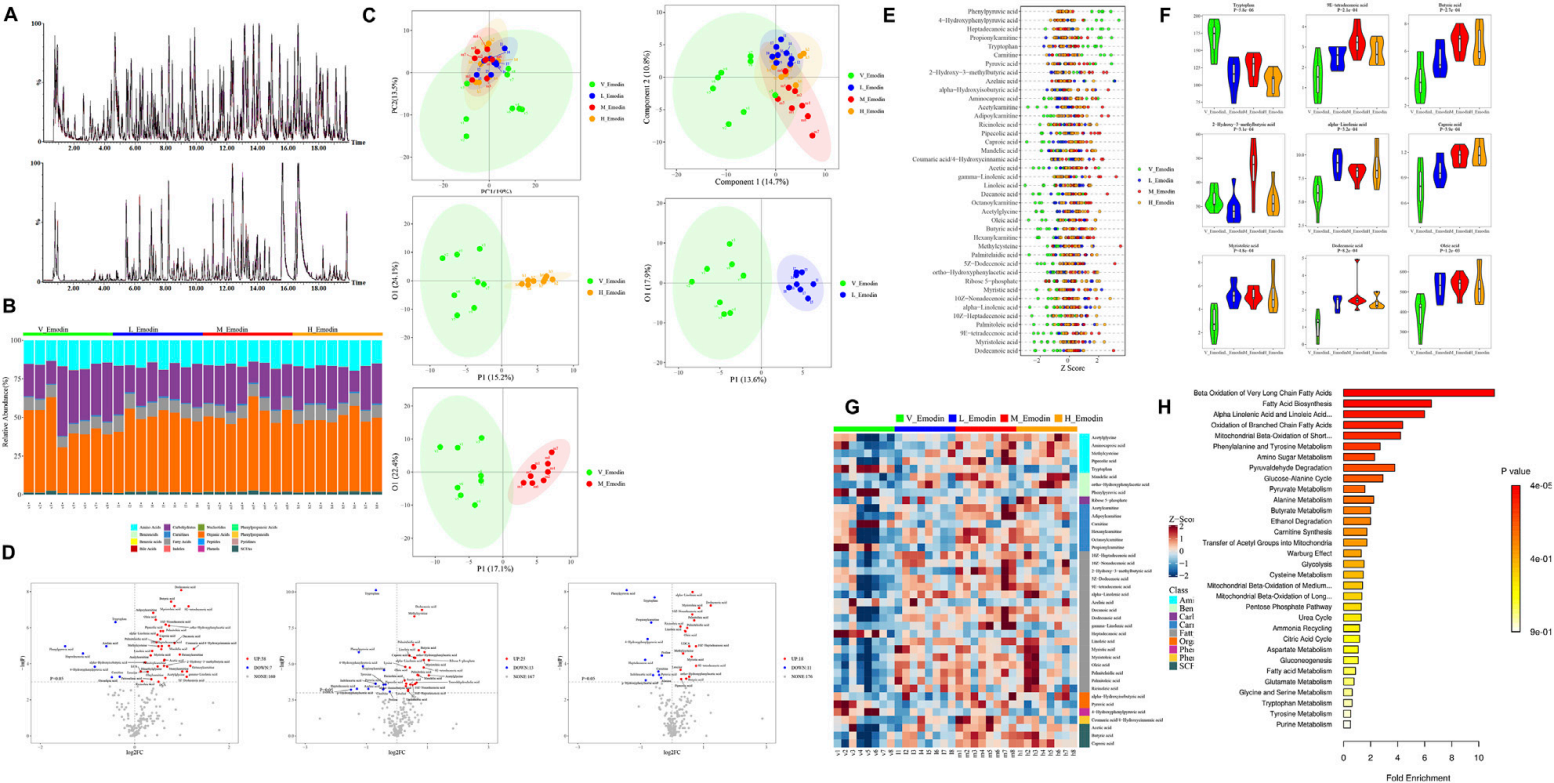
Emodin’s impact on FFAs metabolism in mice: **(A)** Chromatogram of endogenous metabolites in mouse blood analyzed via UPLC/MS/MS; **(B)** phenotypic characterization of various metabolites from samples; **(C)** Multivariate statistical analyses using PCA and OPLS-DA across the four groups; **(D)** Volcano plot illustrating altered metabolites between the four groups; **(E)** Z-core analysis of differential metabolites across the four groups; **(F)** Comparison of the top nine differential metabolites ranked by *p*-value in unidimensional statistical analysis across the four groups; **(G)** Heatmap depicting thirty-nine potential biomarkers associated with emodin’s hepatotoxicity; **(H)** Pathways linked to the endogenous disturbance as predicted by KEGG enrichment analysis. Significance levels are indicated as follows: *p < 0.05, **p < 0.01, ***p < 0.001, and ****p < 0.0001.

Each metabolite was identified based on the chromatographic peak and mass spectrum fragment information obtained through UPLC-MS/MS analyses (Figure 5A). In totally, sixteen types of the endogenous metabolites were identified. The contents of organic acids, sugars, amino acids, FFAs, and SCFAs constituted more than 99% of all compounds. Notably phenols, carnitines, FFAs, SCFAs, and phenylpropanoids exhibited significant differences among the groups (Figure 5B). Additionally, Figure 5C presents the images of PCA, PLS-DA, and OPLS-DA analyses among the four groups. It is evident that the plots generated by these three multivariate statistical methods exhibit clear separation, clustering in a consistent direction. This outcome indicates that the phenotype of endogenous metabolites in mice has been altered following 8 weeks of emodin administration.

Subsequently, a volcanic plot of unidimensional metabolites, with a threshold set as *p <* 0.05 and |log2FC|>0, was generated to identify the differential metabolites (Figure 5D). In comparison to the normal group, the lose-dose emodin group exhibited an upregulation of 18 metabolites (depicted in light red) and a downregulation of 11 metabolites (depicted in light deep blue). The medium-dose emodin group demonstrated an upregulation of 35 metabolites and a downregulation of 7 metabolites. In contrast, the high-dose emodin group showed an upregulation of 25 metabolites and a downregulation of 11 metabolites. Among these metabolites exhibiting significant variation, the nine most prominent differential metabolites, as determined by *p*-value ranking in unidimensional statistical analysis, included tryptophan, methyl trans-myristate, butyric acid, 2-hydroxy-3-methylbutyric acid, hydroxyisobutyric acid, hexanoic acid, myristate acid, lauric acid, and oleic acid (Figures 5E, F). Furthermore, a total of 39 metabolites exhibited significant differences between three treatment groups and the normal group, as detailed in Table 1 and illustrated in Figure 5G. Notably, 21 of these 39 metabolites were FFAs, including both FFAs and SCFAs. This finding suggests that the observed alternations in FFAs and SCFAs are closely associated with disturbance in intestinal microbiota and hepatic lipid accumulation induced by emodin. Subsequent KEGG enrichment analysis revealed that the metabolic changes resulting from varying doses of emodin primarily impact the biosynthesis of unsaturated fatty acid and fatty acid (Figure 5H).

**TABLE 1:** Significant differential metabolites between the normal group and three doses of emodin groups.

Class	Metabolite	Trends in treatments	VIP^a^	Fold change	log2FC	Uni_P^b^
SCFAs	Acetic acid	↑	1.04816	1.35282	0.43597	0.015695
SCFAs	Butyric acid	↑	1.365201	1.640458	0.714099	0.000266
SCFAs	Caproic acid	↑	1.414765	1.479964	0.565562	0.00039
Fatty Acids	Azelaic acid	↓	1.236392	0.737544	−0.4392	0.049859
Fatty Acids	Heptadecanoic acid	↓	1.283559	0.404059	−1.30736	0.027853
Fatty Acids	2-Hydroxy-3-methylbutyric acid	↑	1.720977	0.983788	−0.02358	0.00031
Fatty Acids	Decanoic acid	↑	1.392181	1.419615	0.5055	0.007106
Fatty Acids	5Z-Dodecenoic acid	↑	1.383854	1.641059	0.714627	0.037736
Fatty Acids	Dodecanoic acid	↑	1.392181	1.765357	0.81996	0.000825
Fatty Acids	Myristoleic acid	↑	1.273515	1.838008	0.878143	0.000482
Fatty Acids	9E-tetradecenoic acid	↑	1.351264	1.836225	0.876743	0.000213
Fatty Acids	Ricinoleic acid	↑	1.224836	1.192284	0.253727	0.01345
Fatty Acids	Myristic acid	↑	1.012886	1.454155	0.540181	0.042157
Fatty Acids	Palmitoleic acid	↑	1.193602	1.59433	0.67295	0.003759
Fatty Acids	Palmitelaidic acid	↑	1.233028	1.44424	0.530311	0.001278
Fatty Acids	10Z-Heptadecenoic acid	↑	1.331933	1.429268	0.515276	0.019105
Fatty Acids	alpha-Linolenic acid	↑	1.410748	1.51386	0.598232	0.000324
Fatty Acids	gamma-Linolenic acid	↑	1.358047	1.144651	0.194908	0.048481
Fatty Acids	Linoleic acid	↑	1.251607	1.329359	0.410731	0.007373
Fatty Acids	Oleic acid	↑	1.215408	1.337987	0.420064	0.001196
Fatty Acids	10Z-Nonadecenoic acid	↑	1.178587	1.505915	0.59064	0.007925
Amino Acids	Aminocaproic acid	↑	1.108131	1.438994	0.525061	0.040027
Amino Acids	Methylcysteine	↑	1.034871	1.485496	0.570945	0.004403
Amino Acids	Acetylglycine	↑	1.367266	2.063648	1.045197	0.007239
Amino Acids	Pipecolic acid	↑	1.315760	1.232854	0.302002	0.001559
Amino Acids	Tryptophan	↓	1.597543	0.620614	−0.68823	5.76E-06
Benzenoids	ortho-Hydroxyphenylacetic acid	↑	1.241526	1.872205	0.904738	0.008859
Benzenoids	Phenylpyruvic acid	↓	1.469179	0.421164	−1.24755	0.002313
Benzenoids	Mandelic acid	↑	1.150861	1.630725	0.705514	0.021156
Phenylpropanoids	Coumaric acid/4-Hydroxycinnamic acid	↑	1.313683	1.323672	0.404546	0.016564
Phenols	4-Hydroxyphenylpyruvic acid	↓	1.277047	0.462679	−1.11192	0.015818
Organic Acids	alpha-Hydroxyisobutyric acid	↑	1.155949	1.087604	0.121153	0.028034
Organic Acids	Pyruvic acid	↓	1.469179	0.737239	−0.4398	0.009901
Carnitines	Carnitine	↓	1.523435	0.733341	−0.44744	0.007440
Carnitines	Acetylcarnitine	↑	1.242146	1.153392	0.205882	0.013973
Carnitines	Propionylcarnitine	↓	1.523435	0.747906	−0.41907	0.001785
Carnitines	Hexanylcarnitine	↓	1.27459	1.600882	0.678867	0.007297
Carnitines	Adipoylcarnitine	↓	1.419355	1.156314	0.209534	0.005768
Carnitines	Octanoylcarnitine	↑	1.205436	1.59979	0.677882	0.005725
Carbohydrates	Ribose 5-phosphate	↑	1.54789	2.061258	1.043525	0.015554

^a,b^Significantly differential metabolites with VIP, values >1.0 and *p* < 0.05 were selected.

### 3.6 Validation of the hepatotoxic effects of emodin via the intestinal tract through the implementation of probiotics and fecal transplantation

**FIGURE 6:**
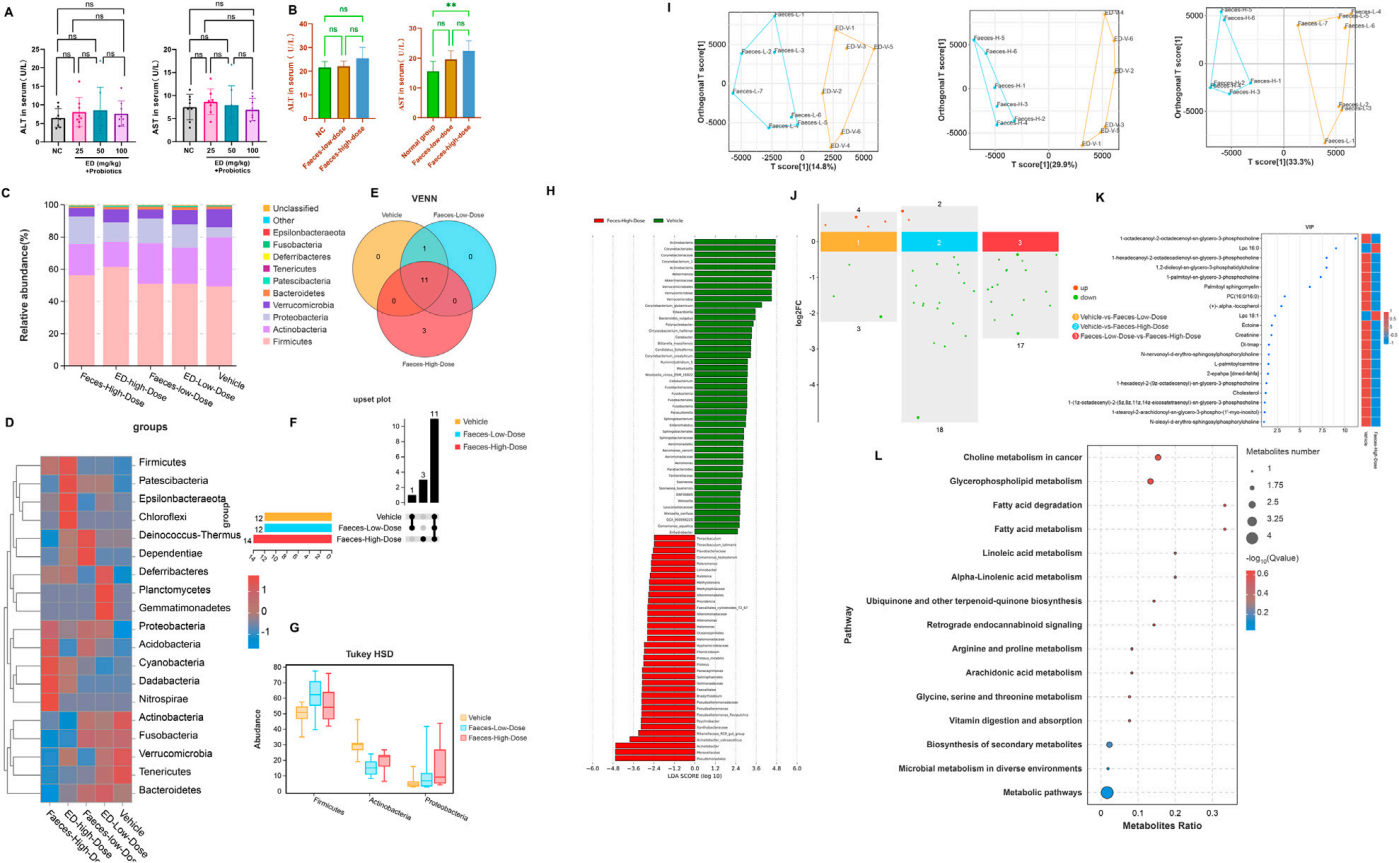
The impact of combined probiotic administration and fecal transplantation on emodin-induced liver injury: **(A)** Comparison of ALT and AST levels among groups receiving probiotics in conjunction with emodin at dosages of 25, 50, and 100 mg/kg/day. **(B)** Comparison of ALT and AST levels among cohorts undergoing fecal transplantation from low-dose and high-dose emodin treatment groups. **(C)** Species distribution histogram of the intestinal microbiota in mice following fecal transplantation. **(D)** The heatmap illustrating the composition of the intestinal microbiota across four distinct treatment groups. **(E)** The Venn analysis of indicator species within the intestinal microbiota of mice following the fecal transplantation. **(F)** The upset plot of the indicator species among groups. **(G)** The potential marker species of the intestinal microbiota at the phylum level with the *p*-value lower than 0.05. **(H)** Lefse anaysis at genus level between the normal group and the Faeces-high-dose group. **(I)** OPLS-DA analysis for the endogenous metabolites between the fecal transplantation groups and the normal group. **(J)** The up and down-regulations of the potential biomarkers with |log2FC|>0. **(K)** The potential biomarkers with the VIP >1.0. **(L)** The bubble map for enrichment pathways with significant differences.

As illustrated in Figure 6A, following an 8-week regimen of combined probiotics and emodin administration, no significant alternations were observed in ALT and AST levels in the treatment group compared to the normal group. Similarity, ALT levels remained unchanged in both faeces-low-dose and faeces-high-dose group (Figure 6B). However, AST levels in the faeces-high-dose group were approximately 1.54 folds higher than those in the normal group, indicating a potential hepatotoxic effect of emodin via the intestines.

Furthermore, 16S rDNA sequencing analysis revealed that the composition of intestinal microflora in mice receiving the fecal transplantation from the lose-dose emodin group was comparable to that observed in the low-dose emodin administration cohort, with no significant differences when compared to the normal group. In contrast, the composition of the microbiota of mice transplanted with faeces from the high-dose emodin group mirrored that of the high-dose emodin administration cohort (Figures 6C, D). Notably, when compared to the normal group, there were significant alternation in microbial phylum within the high-dose faeces transplantation group (Figures 6E, F), characterized by marked increase of *Firmicutes, Actinobacteria* and *Proteobacteria* (Figure 6G). Further analysis using LEfSe analysis revealed significant changes in the genera associated with these three phylum levels between the normal group and the Faeces-High-dose group (Figure 6H). These findings indicate that the impact of transplanted microbiota on the intestinal microecology of mice is fundamentally comparable to that of emodin.

In addition, metabolomics analysis of mice serum from the faeces transplantation groups demonstrated a significant alternation in the metabolite profile compared to the normal group. OPLS-DA effectively differentiated the sample plots corresponding to the Faces-low-dose group, Faeces-high-dose group, and the normal group into two distinct regions (Figure 6I). Based on |log2FC|>0 and VIP>1, twenty differential metabolites were identified between the normal and Faeces-high-dose groups, with 18 metabolites being downregulated and 2 metabolites being upregulated (Figure 6J). The metabolites that exhibited significant alternations were predominantly phospholipids, FFAs, and amino acids (Figure 6K). KEGG enrichment analysis further indicated that the alternations in these metabolites were closely associated with fatty acid degradation and fatty acid metabolism (Figure 6L).

## 4 Discussion

If a substantial quantity of anthraquinones is administered over an extended period, it is anticipated to not only persistently stimulate the intestinal tract, but also induce significant reactions in other organs, particularly the liver. However, the precise effects of varying dose of emodin on the intestinal tract and liver have not been extensively studied. Consequently, the current study selects emodin as a representative anthraquinones to explore its potential hepatotoxic effects mediated through the intestinal tract.

Initially, the animal experiment conducted in this study revealed that varying doses of emodin significantly influenced the mental and physical conditions of the mice. In majority of mice in the treatment groups exhibited characteristic symptoms of intestinal stress, such as irritability, aggression, and diarrhea, which intensified with the prolonged emodin administration. Given the observed variations in these external behaviors, it is imperative to closely examine the internal changes in the mice at the conclusion of the experiment. For instance, the coefficients of the thymus, spleen, and liver exhibited a notable increase while the length of the colon significantly decreased with escalating doses of emodin. Specifically, those organ coefficients in the high-dose group with 100 mg/kg/day emodin were nearly double those observed in the normal group, whereas the colon length was reduced to approximately half of that in the normal group. These observation suggest that prolonged administration of emodin may induce a systemic immune response and intestinal injury in mice.

Secondly, the animal study revealed that the intestinal injury occurred after an 8-week administration of emodin, which may result in the damage to the intestinal mucosal barrier. This barrier comprises several protective layers, including the inner layer and the outer layers of the intestinal microbiota (An et al., 2022). The inner layer consists of goblet cells and epithelial cells, which produces a substantial amount of antibacterial proteins to maintain the host homeostasis (de Medina et al., 2014). In this study, the experimental findings demonstrated that three doses of emodin inhibited the secretion of acidic mucus by the intestinal goblet cells, hereby impairing the formation of the mucus layer and reducing the content of MUC-2, this notably resulted in the dysfunction of the intestinal mucosal barrier. Concurrently, the administration of three doses of emodin significantly downregulated the expression levels of the tight junction proteins ZO-1 and Occludin in intestinal epithelial cells, suggesting that emodin may further compromise the integrity of the intestinal epithelial cells, hereby to increasing the permeability of the intestinal mucosal barrier.

Furthermore, the intestinal microbiota inhabiting the outer mucosal layer forms a protective membrane on the intestinal epithelium, collaborating with other barriers to jointly resist the invasion of pathogenic bacteria and foreign harmful substances (Thaiss et al., 2016). However, when the gut microbiota becomes imbalanced, due to factors such as an unhealthy diet or prolonged drug exposure, the microbial barrier is compromised, leading to an increase in “bad” opportunistic pathogenic bacteria in the outer layer (Wang et al., 2021). The findings of the current study have demonstrated that emodin significantly increase LPS content of hepatoenteric circulation in colon, blood, and liver. The 16S rDNA sequencing analysis also revealed that administering three doses of emodin significantly altered the diversity and composition of the intestinal microbiota. Specifically, the *Firmicutes* to *Bacteroides* ratio increased markedly with higher doses of emodin at the phylum level. This ratio has emerged as a critical indicator of intestinal diseases (Magne et al., 2020). For instance, several studies have reported that the most consistent alternation observed in patients with NAFLD, as compared to healthy individuals, or between patients with mild and severe NAFLD, is an increase in the relative abundance of *Firmicutes* and a decrease in the abundance of *Bacteroides* (Hu et al., 2020; Zeng and Schnabl, 2022; Campagnoli et al., 2022). In the current study, *Firmicutes* to *Bacteroides* ratio in the medium- and high-dose emodin groups was observed to be twice that of the normal group. This finding provides significant evidence of intestinal microbiota dysbiosis, which is closely linked to intestinal barrier dysfunction resulting from prolonged emodin use. Furthermore, the study also revealed that the administration of emodin led to a marked increase in the relative abundance of *Proteobacteria* at the phylum level. *Proteobacteria*, a class of gram-negative bacteria, are prevalent in the intestinal tract, and their abundance often increases when the host metabolism disrupted (Umeda et al., 2023). In this study, a reduction in beneficial bacteria, coupled with an increase in gram-negative bacteria, signifies an elevated source of LPS in mice administered with 50 or 100 mg/kg/day of emodin over an 8-week period. Concurrently, emodin induced intestinal barrier dysfunction enhances the permeability of excessive LPS through the intestinal mucosal barrier. All of these factors combined to lead to liver damage.

Subsequent pathological analysis verified that administering emodin at doses of 50 or 100 mg/kg/day over an 8-week period resulted in pronounced significant steatosis and inflammatory damage in the hepatic tissue of mice. Specifically, there was a significant elevation in plasma levels of AST and ALT. Histological examination of liver section stained with HE revealed prominent adipose vacuoles and inflammatory cell infiltration following the administration of 50 or 100 mg/kg/day for 8 weeks. It is noteworthy that the increase in FFAs and the dysbiosis of intestinal flora are both critical factors closely associated with excessive lipid accumulation in the liver. The dysregulation of intestinal microbiota can specifically activate FFAs as nutrient sensing receptor (Kåhrström et al., 2016). When there is an excessive influx of FFAs to the liver, it facilitates the synthesis of triglycerides, leading to hepatic lipid accumulation (Holzhütter and Berndt, 2021). In the current study, administration of emodin remarkably increased the levels of TC and TG in the plasma of mice. Concurrently, oil red O staining also showed adipocyte infiltration in the liver tissue of mice. These findings demonstrate that the emodin doses employed in this study may contribute to enhanced lipid deposition in the liver to a certain extent. Simultaneously, the blood metabolomics analysis revealed that three doses of emodin significantly modulated the levels of 3 types of SCFAs, namely acetic acid, butyric acid, and caproic acid, as well as 18 types of FFAs in mice. It is noteworthy that considerable attention should be direct towards the remarkable alternations in SCFAs and FFAs. On one hand, SCFAs play a critical role in regulating the differentiation and polarization of T cells, thereby maintaining intestinal immune homeostasis (Wang et al., 2023). On the other hand, SCFAs can both inhibit and promote neutrophil activity and influence the immune regulation of monocytes and macrophages (Vinolo et al., 2011). Several studies have demonstrated that the elevated concentrations of acetic acid and butyric acid in obese mice are associated with a significant increase in the proportion of *Firmicutes* and *Bacteroides* (Schwiertz et al., 2010; Liu et al., 2018; Guo et al., 2022). Supporting these findings, another study indicated that the acetic acid serves as a substrate for adipogenesis, thereby promoting fat deposition in mice (Luo et al., 2018). In the current study, a similar effect was observed, where varying doses of emodin exhibited a marked increase in acetic acid level and a substantial rise in the *Firmicutes* and *Bacteroides* ratio. Furthermore, since the present study found that prolonged administration of emodin significantly elevated the levels of 18 types of FFAs in the serum of mice. This elevation is a critical factor contributing to the development of hepatic steatosis. Collectively, these findings suggested that the dysregulation of fatty acid metabolism, encompassing both FFAs and SCFAs, may constitute a fundamental pathological mechanism underlying emodin-induced liver injury.

Notably, the TLR4-MyD88-NF-κB signaling pathway serves as an critical nexus associated with hepatic tissue infection, injury, and inflammation (Jia et al., 2014). Upon activation of the TLR4 signaling pathway, a protein complex comprising MyD88 and NF-κB is assembled, facilitating the proteolytic processing of activated NF-κB (Zhou et al., 2018). This process subsequently induces the maturation and secretion of pro-inflammatory cytokines including TNF-α, IL-1β, IL-18, and others, thereby establishing a localized inflammatory micro environment within liver tissue (Jagavelu et al., 2010). The findings of this study demonstrated a clear upregulation in the mRNA levels of TNF-α, IL-1β, IL-6, and IL-18. Concurrently, there is an observed increase in both mRNA levels and proteins expression of TLR4, MyD88, and p-65, which correlates with escalating doses of emodin. The study demonstrated a dose-dependent increase in mRNA levels of inflammatory cytokines in the hepatic tissue of animals, corresponding with escalating doses of emodin. Furthermore, the upregulation of these inflammatory mediators is associated with the onset of insulin resistance, subsequently resulting in dysregulated lipid metabolism within the liver (Alper et al., 2016). These findings suggested that emodin significantly elevates pro-inflammatory cytokine levels, thereby inducing hepatic inflammatory responses and disturbances in lipid metabolism. The elevation of free fatty acids serves as an intermediary factor in the development and the accumulation of hepatic fat deposits.

**FIGURE 7:**
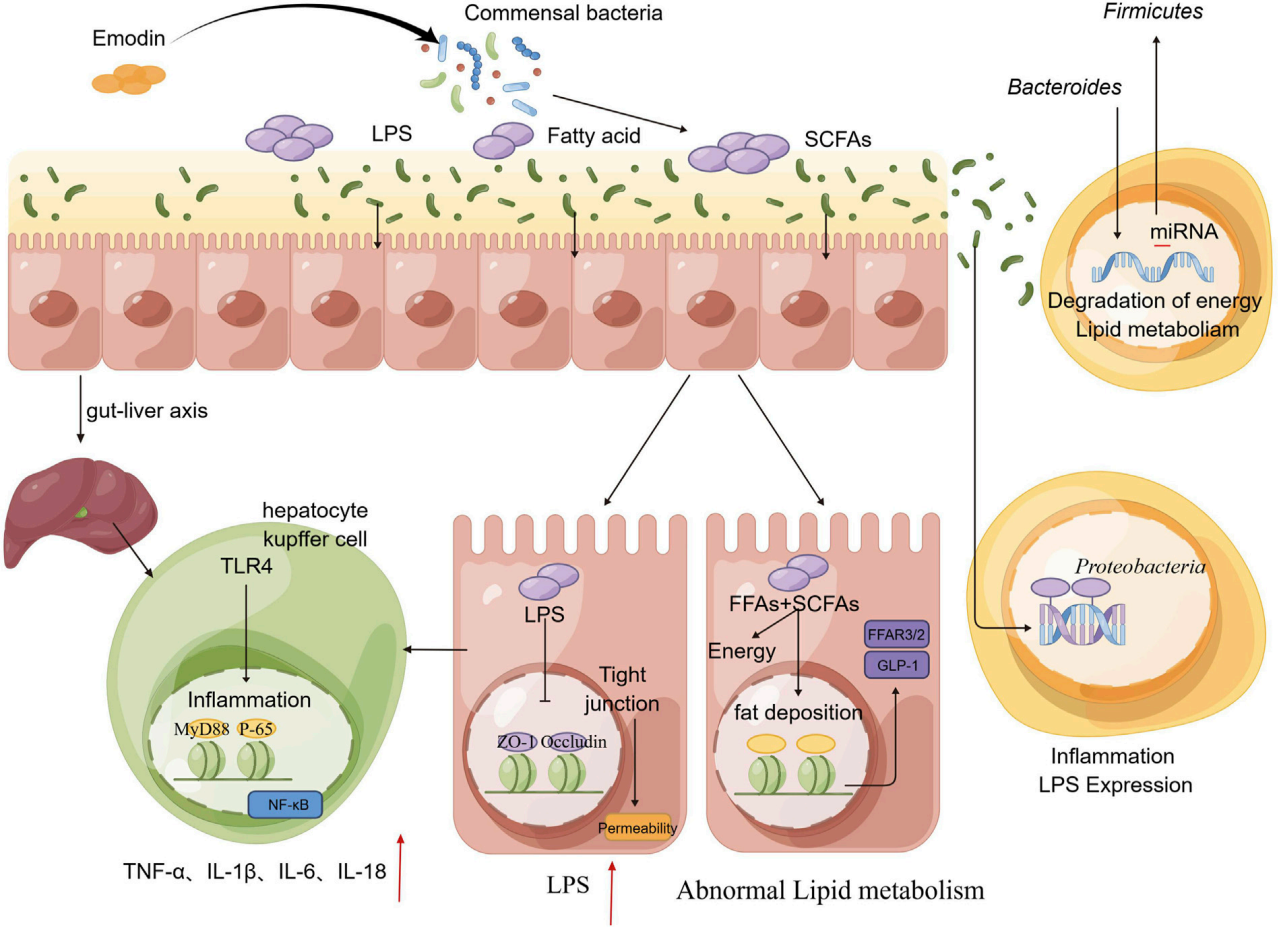
A potential road-map detailed the injury to the intestinal tract and liver induced by emodin.

In this study, we initially observed that varying doses of emodin disrupted the equilibrium of the intestinal microbiota in mice and compromised the intestinal barrier through inflammatory process. This disruption was associated with increased permeability and elevated LPS levels, which in turn could participate hepatic fat accumulation and inflammation. Subsequently, following an 8-week regimen combining the probiotics and emodin, we found no significant alternations in hepatic function indices like ALT and AST. This finding indicates that the probiotics effectively mitigate the liver damage induced by the high-dose emodin. Numerous clinical trials and vivo experiments have significantly advanced our understanding of the critical roles that probiotics play in diseases associated with the human gut microbiome as well as documented that probiotics can modulate the intestinal microbiota, potentially contributing to the management of various bowel diseases and the enhancement of overall health (Abraham and Quigley, 2017; Kim et al., 2019). Then, we conducted additional investigations to elucidate the role of emodin in inducing liver injury via the intestinal tract by employing the fecal microbiota transplantation. The results indicated that the overall condition of mice receiving the fecal transplantation from the low-dose emodin group was comparable to that of the normal group, with no significant symptoms of diarrhea observed. Conversely, mice transplanted with fecal matter from the high-dose emodin group exhibited delayed onset of diarrhea compared to those directly administered high-dose emodin. However, varying degrees of diarrhea were observed in these mice after 4–5 weeks post-microbiota transplantation. Subsequent microbiota sequencing demonstrated notable alternations in the microbiota of mice receiving transplants of feces containing high-dose emodin. These alternations were characterized by significant increases in the relative abundance of *Firmicutes* and *Proteobacteria,* align with the microbiota changes observed in mice administered high-dose of emodin. In additional, metabolomic analysis for the serum of these mice from the Faeces-high-dose group revealed marked differences in serum metabolites compared to the normal group, primarily manifesting as disruptions in phospholipid and fatty acid metabolism. Concurrently, the substantial elevation in AST levels further corroborated that microbial and metabolic disturbance induced by high-dose of emodin can lead to liver damage. Based on the forementioned experimental results, it is tentatively hypothesized that emodin primarily induces an increase in *Firmicutes* and a decrease in *Bacteroides,* leading to the production of additional LPS, FFAs, and SCFAs. These excessive pathogens may activate intestinal epithelial cells, resulting in disruption of tight junctions and the impairment of the intestinal barriers. Consequently, increased LPS can more readily permeate into the liver via the gut-liver axis, thereby activating TLR4 signal pathway and triggering an inflammatory response. Simultaneously, a greater quantity of FFAs is transferred into bloodstream from the intestinal mucosal barrier, subsequently disrupting the synthesis and breakdown of triglycerides, which leads to lipid accumulation in the liver (Figure 7). Collectively, in this study, a long-term toxicity experiment was conducted to examine the deleterious effects of emodin on murine hepatic tissue. Emodin was administered to mice at varying dosages (0, 25, 50, 100 mg/kg/day) over an 8-week period to assess its toxicological impact on both the colon and liver. The hepatotoxic effects of emodin were confirmed through a series of experiments, which established a foundation for the rational clinical use of emodin-containing herbs, such as Polygoni Multiflori Radix. Nevertheless, further investigation is required to elucidate the underlying mechanisms of the observed experimental phenomena.

## 5 Conclusion

At higher doses (50 or 100 mg/kg/day), emodin has the potential to compromise the integrity of the intestinal mucosal barrier, leading to increased permeability to LPS and FFAs, which may subsequently induce fatty liver hepatitis in murine models.

## Data Availability

The raw data supporting the study are publicly available. The raw 16 S rDNA sequencing data for gut microbiota that support the present findings of this study are openly available in NCBI, accession number PRJNA1163986; available at https://www.ncbi.nlm.nih.gov/sra/PRJNA1163986.

## References

[B1] Abraham B. P. Quigley E. M. M. (2017). Probiotics in inflammatory bowel disease. Gastroenterology Clin. N. Am. 46 (4), 769–782. 10.1016/j.gtc.2017.08.003 29173520

[B2] Alper B. Erdogan B. Erdogan M. Ö. Bozan K. Can M. (2016). Associations of trauma severity with mean platelet volume and levels of systemic inflammatory markers (IL1β, IL6, TNFα, and CRP). Mediat. Inflamm. 2016, 9894716. 10.1155/2016/9894716 PMC4835648 27127347

[B3] An J. Liu Y. Wang Y. Fan R. Hu X. Zhang F. (2022). The role of intestinal mucosal barrier in autoimmune disease: a potential target. Front. Immunol. 13, 871713. 10.3389/fimmu.2022.871713 35844539 PMC9284064

[B4] Campagnoli L. I. M. Marchesi N. Vairetti M. Pascale A. Ferrigno A. Barbieri A. (2022). Age-related NAFLD: the use of probiotics as a supportive therapeutic intervention. Cells 11 (18), 2827. 10.3390/cells11182827 36139402 PMC9497179

[B5] Dai Y. Jia Z. Fang C. Zhu M. Yan X. Zhang Y. (2023). Polygoni Multiflori Radix interferes with bile acid metabolism homeostasis by inhibiting Fxr transcription, leading to cholestasis. Front. Pharmacol. 14, 1099935. 10.3389/fphar.2023.1099935 36950015 PMC10025474

[B6] de Medina F. Romero-Calvo I. Mascaraque C. Martínez-Augustin O. (2014). Intestinal inflammation and mucosal barrier function. Inflamm. bowel Dis. 20 (12), 2394–2404. 10.1097/mib.0000000000000204 25222662

[B7] Elsohly M. A. Gul W. Avula B. Khan I. A. (2007). Determination of the anthraquinones aloe-emodin and aloin-A by liquid chromatography with mass spectrometric and diode array detection. J. AOAC Int. 90 (1), 28–42. 10.1093/jaoac/90.1.28 17373434

[B8] Engelmann C. Sheikh M. Sharma S. Kondo T. Loeffler-Wirth H. Zheng Y. B. (2020). Toll-like receptor 4 is a therapeutic target for prevention and treatment of liver failure. J. hepatology 73 (1), 102–112. 10.1016/j.jhep.2020.01.011 31987990

[B9] Guo J. Zhang M. Wang H. Li N. Lu Z. Li L. (2022). Gut microbiota and short chain fatty acids partially mediate the beneficial effects of inulin on metabolic disorders in obese ob/ob mice. J. food Biochem. 46 (5), e14063. 10.1111/jfbc.14063 35128673

[B10] He X. Liu J. Long G. Xia X. H. Liu M. (2021). 2,3,5,4'-Tetrahydroxystilbene-2-O-β-D-glucoside, a major bioactive component from Polygoni multiflori Radix (Heshouwu) suppresses DSS induced acute colitis in BALb/c mice by modulating gut microbiota. Biomed. and Pharmacother. 137, 111420. 10.1016/j.biopha.2021.111420 33761623

[B11] Holzhütter H. G. Berndt N. (2021). Computational hypothesis: how intra-hepatic functional heterogeneity may influence the cascading progression of free fatty acid-induced non-alcoholic fatty liver disease (NAFLD). Cells 10 (3), 578. 10.3390/cells10030578 33808045 PMC7999144

[B12] Hu H. Lin A. Kong M. Yao X. Yin M. Xia H. (2020). Intestinal microbiome and NAFLD: molecular insights and therapeutic perspectives. J. gastroenterology 55 (2), 142–158. 10.1007/s00535-019-01649-8 PMC6981320 31845054

[B13] Hu Y. H. Li D. K. Quan Z. Y. Wang C. Y. Zhou M. Sun Z. X. (2021). Exploration of components and mechanisms of Polygoni Multiflori Radix-induced hepatotoxicity using siRNA-mediated CYP3A4 or UGT1A1 knockdown liver cells. J. Ethnopharmacol. 270, 113845. 10.1016/j.jep.2021.113845 33485974

[B14] Jagavelu K. Routray C. Shergill U. O'Hara S. P. Faubion W. Shah V. H. (2010). Endothelial cell toll-like receptor 4 regulates fibrosis-associated angiogenesis in the liver. Hepatol. Baltim. Md. 52 (2), 590–601. 10.1002/hep.23739 PMC2916032 20564354

[B15] Jia L. Vianna C. R. Fukuda M. Berglund E. D. Liu C. Tao C. (2014). Hepatocyte Toll-like receptor 4 regulates obesity-induced inflammation and insulin resistance. Nat. Commun. 5, 3878. 10.1038/ncomms4878 24815961 PMC4080408

[B16] Kåhrström C. T. Pariente N. Weiss U. (2016). Intestinal microbiota in health and disease. Nature 535 (7610), 47. 10.1038/535047a 27383978

[B17] Kim S. K. Guevarra R. B. Kim Y. T. Kwon J. Kim H. Cho J. H. (2019). Role of probiotics in human gut microbiome-associated diseases. J. Microbiol. Biotechnol. 29 (9), 1335–1340. 10.4014/jmb.1906.06064 31434172

[B18] Liang Z. Chen H. Yu Z. Zhao Z. (2010). Comparison of raw and processed Radix Polygoni Multiflori (Heshouwu) by high performance liquid chromatography and mass spectrometry. Chin. Med. 5, 29. 10.1186/1749-8546-5-29 20704710 PMC2930642

[B19] Liu B. Wang W. Zhu X. Sun X. Xiao J. Li D. (2018). Response of gut microbiota to dietary fiber and metabolic interaction with SCFAs in piglets. Front. Microbiol. 9, 2344. 10.3389/fmicb.2018.02344 30323803 PMC6172335

[B20] Luo X. Li H. Ma L. Zhou J. Guo X. Woo S. L. (2018). Expression of STING is increased in liver tissues from patients with NAFLD and promotes macrophage-mediated hepatic inflammation and fibrosis in mice. Gastroenterology 155 (6), 1971–1984.e4. 10.1053/j.gastro.2018.09.010 30213555 PMC6279491

[B21] Magne F. Gotteland M. Gauthier L. Zazueta A. Pesoa S. Navarrete P. (2020). The firmicutes/bacteroidetes ratio: a relevant marker of gut dysbiosis in obese patients? Nutrients 12 (5), 1474. 10.3390/nu12051474 32438689 PMC7285218

[B22] Qian G. Leung S. Y. Lu G. Leung K. S. (2008). Optimization and validation of a chromatographic method for the simultaneous quantification of six bioactive compounds in Rhizoma et Radix Polygoni Cuspidati. J. Pharm. Pharmacol. 60 (1), 107–113. 10.1211/jpp.60.1.0014 18088511

[B23] Schneider K. M. Mohs A. Gui W. Galvez E. J. C. Candels L. S. Hoenicke L. (2022). Imbalanced gut microbiota fuels hepatocellular carcinoma development by shaping the hepatic inflammatory microenvironment. Nat. Commun. 13 (1), 3964. 10.1038/s41467-022-31312-5 35803930 PMC9270328

[B24] Schwiertz A. Taras D. Schäfer K. Beijer S. Bos N. A. Donus C. (2010). Microbiota and SCFA in lean and overweight healthy subjects. Obes. (Silver Spring, Md.) 18 (1), 190–195. 10.1038/oby.2009.167 19498350

[B25] Semwal R. B. Semwal D. K. Combrinck S. Viljoen A. (2021). Emodin-A natural anthraquinone derivative with diverse pharmacological activities. Phytochemistry 190, 112854. 10.1016/j.phytochem.2021.112854 34311280

[B26] Thaiss C. A. Zmora N. Levy M. Elinav E. (2016). The microbiome and innate immunity. Nature 535 (7610), 65–74. 10.1038/nature18847 27383981

[B27] Umeda S. Sujino T. Miyamoto K. Yoshimatsu Y. Harada Y. Nishiyama K. (2023). D-Amino acids ameliorate experimental colitis and cholangitis by inhibiting growth of Proteobacteria: potential therapeutic role in inflammatory bowel disease. Cell. Mol. gastroenterology hepatology 16 (6), 1011–1031. 10.1016/j.jcmgh.2023.08.002 PMC10632532 37567385

[B28] Vinolo M. A. Rodrigues H. G. Nachbar R. T. Curi R. (2011). Regulation of inflammation by short chain fatty acids. Nutrients 3 (10), 858–876. 10.3390/nu3100858 22254083 PMC3257741

[B29] Wang H. H. (1993). Antitrichomonal action of emodin in mice. J. Ethnopharmacol. 40 (2), 111–116. 10.1016/0378-8741(93)90055-a 8133650

[B30] Wang J. Zhu N. Su X. Gao Y. Yang R. (2023). Gut-microbiota-derived metabolites maintain gut and systemic immune homeostasis. Cells 12 (5), 793. 10.3390/cells12050793 36899929 PMC10000530

[B31] Wang R. Tang R. Li B. Ma X. Schnabl B. Tilg H. (2021). Gut microbiome, liver immunology, and liver diseases. Cell. and Mol. Immunol. 18 (1), 4–17. 10.1038/s41423-020-00592-6 33318628 PMC7852541

[B32] Wang S. Kong X. Chen N. Hu P. Boucetta H. Hu Z. (2022). Hepatotoxic metabolites in Polygoni multiflori Radix- comparative toxicology in mice. Front. Pharmacol. 13, 1007284. 10.3389/fphar.2022.1007284 36304159 PMC9592908

[B33] Xia X. H. Yuan Y. Y. Liu M. (2017). The assessment of the chronic hepatotoxicity induced by Polygoni Multiflori Radix in rats: a pilot study by using untargeted metabolomics method. J. Ethnopharmacol. 203, 182–190. 10.1016/j.jep.2017.03.046 28365236

[B34] Xing Y. Wang L. Wang C. Zhang Y. Zhang Y. Hu L. (2019). Pharmacokinetic studies unveiled the drug-drug interaction between trans-2,3,5,4'-tetrahydroxystilbene-2-O-β-d-glucopyranoside and emodin that may contribute to the idiosyncratic hepatotoxicity of Polygoni Multiflori Radix. J. Pharm. Biomed. analysis 164, 672–680. 10.1016/j.jpba.2018.11.034 30472586

[B35] Yang C. Wang S. Guo X. Sun J. Liu L. Wu L. (2014). Simultaneous determination of seven anthraquinones in rat plasma by Ultra High Performance Liquid Chromatography-tandem Mass Spectrometry and pharmacokinetic study after oral administration of Semen Cassiae extract. J. Ethnopharmacol. 169, 305–313. 10.1016/j.jep.2015.04.008 25907980

[B36] Zeng S. Schnabl B. (2022). Roles for the mycobiome in liver disease. Liver Int. official J. Int. Assoc. Study Liver 42 (4), 729–741. 10.1111/liv.15160 PMC8930708 34995410

[B37] Zhou P. She Y. Dong N. Li P. He H. Borio A. (2018). Alpha-kinase 1 is a cytosolic innate immune receptor for bacterial ADP-heptose. Nature 561 (7721), 122–126. 10.1038/s41586-018-0433-3 30111836

